# A multidisciplinary Delphi consensus on the modern definition of pruritus: Sensation and disease

**DOI:** 10.1111/jdv.20851

**Published:** 2025-07-17

**Authors:** Sonja Ständer, Martin Schmelz, Ethan Lerner, Hiroyuki Murota, Leigh Nattkemper, Adam Reich, Lea‐Sophie Stahl, Gil Yosipovitch, Elke Weisshaar, Henning Wiegmann, Christian Apfelbacher, Brian S. Kim

**Affiliations:** ^1^ Department of Dermatology and Center for Chronic Pruritus University Hospital Münster Münster Germany; ^2^ Institut für Experimentelle Schmerzforschung Medizinische Fakultät Mannheim, Universität Heidelberg Mannheim Germany; ^3^ Cutaneous Biology Research Center, Massachusetts General Hospital Charlestown Massachusetts USA; ^4^ Department of Dermatology Nagasaki University Graduate School of Biomedical Sciences Nagasaki Japan; ^5^ Miami Itch Center, Dr. Phillip Frost Department of Dermatology and Cutaneous Surgery University of Miami Miller School of Medicine Miami Florida USA; ^6^ Department of Dermatology University of Rzeszow Rzeszów Poland; ^7^ Universitätsklinikum Heidelberg, Klinik für Dermatologie / Sektion Berufsdermatologie Heidelberg Germany; ^8^ Institute of Social Medicine and Health Systems Research Medical Faculty, Otto von Guericke University Magdeburg Germany; ^9^ Kimberly and Eric J. Waldman Department of Dermatology The Mount Sinai Hospital New York New York USA

**Keywords:** definition, Delphi, itch, pruritus, scratching, skin sensation

## Abstract

**Background:**

The definition of pruritus (synonym: itch) dates back to the 17th century and does not address the complexity of the sensation or the nature of pruritus as a disease.

**Objectives:**

Elaborate a new definition of pruritus comprising emotional aspects, stages and causal attributes.

**Methods:**

In a pre‐Delphi phase, stakeholders from pruritus‐relevant global societies and patients elaborated itch definition proposals. In a Delphi phase, consensus was achieved when ≥75% of the voting participants agreed (7–9 Likert scale rating range) on a statement.

**Results:**

In two Delphi rounds, two statements defining the itch sensation and nine defining chronic pruritus as a disease achieved consensus. The definition of pruritus as an unpleasant sensation commonly triggering an urge to scratch mentions that external or internal factors can trigger, worsen, or improve pruritus. Chronic pruritus is described as a symptom of diseases but also as an independent disease. The definition of chronic pruritus includes temporal, physiological, causal, and quality of life aspects.

**Conclusions:**

The new definition of pruritus allows the sensation to be separated from a disease state and aims to support practical use, including communication with patients, health authorities, and other stakeholders.

## Graphical abstract

Stakeholders from pruritus‐relevant global societies and patients consented to a new definition of pruritus through a Delphi process. The new definition allows the sensation to be separated from the chronic disease state. It considers symptoms and time course, as well as emotional impact and causal aspects.

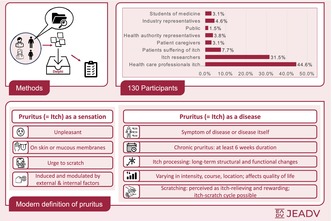

**Why was the study undertaken?**

The current definition of pruritus lacks information on symptoms, stage, emotional and causal attributes.

**What does this study add?**

Acute and chronic pruritus, as well as pruritus as a disease symptom or a disease on its own, including temporal, physiological, causal and quality of life aspects, are defined.

**What are the implications of this study for disease understanding and/or clinical care?**

The new pruritus definition is expected to improve communication with patients, health authorities and other stakeholders and to facilitate the development of itch‐specific therapies independent of the initial trigger.

## INTRODUCTION

Itch or pruritus was first defined by Samuel Haffenreffer in 1660 as *an unpleasant sensation leading to the desire to scratch*.^1^ This definition is still used today.^2^ However, it does not reflect its complexity and its impact on the patient.^3^ Nowadays, the definition of a sensory experience should include symptoms, stages and causal as well as emotional attributes,^4^, ^5^, ^6^ which are essentially missing from the current definition. This weakness was debated during the annual meeting of the International Dermatology Outcome Measures (IDEOM, chair: A. Gottlieb) organization in June 2022.^7^ Patients missed the emotional component referring to quality of life. Considering the high prevalence of pruritus (up to 25% to 40%) in the general population and its impact on quality of life, a more precise definition of the sensation appeared necessary.^8^, ^9^ Consequently, an initiative was formed to reach a consensus on a new definition of human‐experienced itch using the experience of global itch experts, health authority members and patients through a Delphi process.

## METHODS

### Steering committee and participants

The initiative was led by the head of the IDEOM itch group and his deputy (B.K. and S.ST.), who established a steering committee (*n* = 9) of chairs and presidents of the globally relevant itch societies: the International Forum for the Study of Itch (IFSI: E.W., E.L.), the Task Force Pruritus of the European Academy of Dermatology and Venerology (EADV: A.R.), the former Itch Group of the International Association for the Study of Pain (IASP: M.S., G.Y.) and the Japanese Society of Itch (JSI: H.M.). C.A. was invited as an expert in methodology. The steering committee was responsible for selecting participants, developing itch definition statements, monitoring and decision‐making.

A panel of experts meeting strict eligibility criteria, such as pruritus society‐dependent clinical, research and legal experts, patients affected by chronic pruritus (CP) and interested health authority representatives, was invited and participated in voting, providing feedback and consensus‐building (Table S1).

Pre‐Delphi and Delphi approach.

The process was divided into a pre‐Delphi and a Delphi period. The pre‐Delphi period was an open, unstructured exchange for collecting statements regarding the definition of itch. The Delphi method was used as an iterative, structured, anonymous process with the possibility of statistical aggregation of the group response to collect all votes and opinions of a large panel of participants. The advantage of this method is that the anonymity of the participants limits the direct influence of ‘key opinion leader’ participants.

**FIGURE 1:**
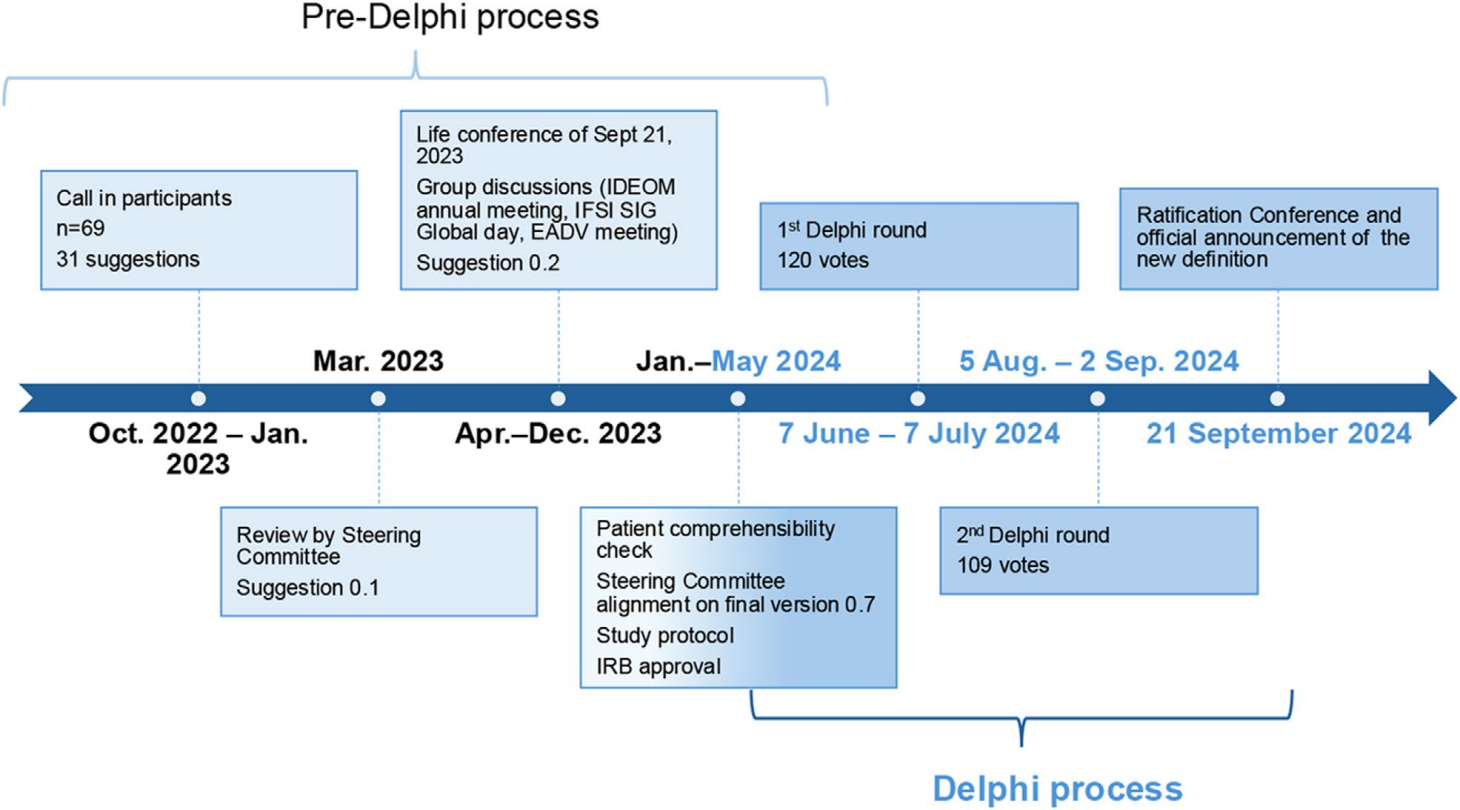
Itch definition Pre‐Delphi and Delphi process. EADV, European Academy of Dermatology and Venerology; IDEOM, International Dermatology Outcome Measures organization; IFSI SIG, International Forum for the Study of Itch Special Interest Group; IRB, Institutional Review Board.

In a pre‐Delphi process (Figure 1), participants registered and entered open suggestions on a digital platform (umfragen.uni‐muenster.de) anonymously. Based on these suggestions, the steering committee created the first set of statements for the itch definition. Subsequently, the statements were discussed with all registered stakeholders, including patients and a broader expert audience of the corresponding society conferences: IDEOM meeting in June 2023 (lead by L.N.), digital IDEOM‐organized conference in September 2023 with a preceding digital poll of registered stakeholders, EADV taskforce meeting in October 2023 and the IFSI SIG meeting in November 2023. Based on the feedback, the definition statements were further modified and aligned within the steering committee. A final check for comprehensibility of the statements was performed with 19 CP patients from Europe (Germany, Greece, Luxembourg, Netherlands, United Kingdom), North America (Canada, USA) and Australia recruited with the help of the societies and the patient organization GlobalSkin. The steering committee then agreed on a final version of the statements.

The Delphi process was performed according to the ‘Reporting guidelines for Delphi techniques in health sciences’^10^ and initiated by applying a predefined protocol (written by L.S.S., S.ST., C.A.). The statements with the respective pro and contra arguments and the question regarding the participants' agreement were programmed in a web‐based survey (www.edelphi.org) by H.W., who organized the Delphi voting without self‐voting. The participants anonymously selected the level of agreement on a 10‐point Likert scale (from 0 = strongly disagree to 9 = strongly agree) and could comment in a free text field. All steering committee members then invited their associates to join the Delphi process. In addition, all previously registered stakeholders were invited directly. The invitees received, along with the invitation, the current statements with the corresponding pro and contra arguments and operating instructions for the edelphi platform. The first Delphi round occurred from 7 June to 7 July 2024, and the second from 5 August to 2 September 2024.

### Data analysis

The data collected were transferred to the latest version of Microsoft Excel and analysed descriptively based on the percentages of participation and agreement. Consensus was achieved when ≥75% of votes were in the 7–9 Likert scale rating range. The consensus was summarized and shared with all participants. Subsequently, the results were presented at the ratification conference. The Ethics Committee of Westfalen‐Lippe, Münster, Germany, approved the study (No. 2024‐045‐f‐S).

## RESULTS

**FIGURE 2:**
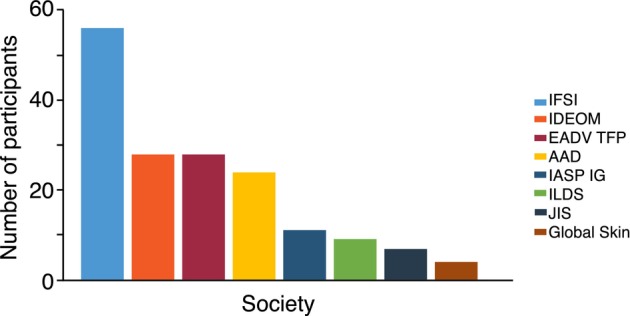
Membership distribution across Delphi participants regarding the organizing societies. Not all participants were members of the organizing or other societies. AAD, American Academy of Dermatology; EADV TFP, European Academy of Dermatology and Venerology Task Force Pruritus; GlobalSkin, International alliance of dermatology patient organizations; IASP IG, International Association for the Study of Pain Itch Group; IDEOM, International Dermatology Outcome Measures organization; IFSI, International Forum for the Study of Itch; ILDS, International League of Dermatological Societies; JIS, Japanese Society of Itch.

In the pre‐Delphi phase (Figure 1), 69 participants (eTable 1) made 31 suggestions, of which the steering committee concluded on a twofold definition: one as a human sensation of the skin and one as a disease. Two statements of the definition as a sensation with variants (1a‐e and 2, Table 1) and nine (3–11, Table 1) of the definition as disease entered the first Delphi round. Of the 180 invited participants, 130 (72.2%) participants in total from multiple societies (Figure 2, eTable 1) voted in two rounds (round 1: *n* = 120, round 2: *n* = 109); 12 (6.7%) rejected the participation, and 38 (21.1%) did not finish the survey. Of the participants, 58 (44.6%) were healthcare professionals, 41 (31.5%) researchers, 10 (7.7%) patients, 4 (3.1%) patient caregivers, 5 (3.8%) from the health authority, 2 (1.5%) from the public, 6 (4.6%) from the industry and 4 (3.1%) students. The participant representation was very similar in both rounds.

**TABLE 1:** Statements for pruritus as a sensation and disease presented at the first and second Delphi rounds and corresponding agreeing voting results.

#	Definition of pruritus as a sensation (*valid for acute and chronic pruritus*)	Voting results No. agreeing (7–9 Likert scale range)/No. voted (%) [mode; IQR]	
		1st Delphi, Total *n* = 120	2nd Delphi, Total *n* = 109
1a	Pruritus (itch) is an unpleasant sensation of the skin and/or neighbouring mucous membranes^a^ commonly triggering an urge to scratch.	84/110 (76.36) [8;1]	NA
1b	Pruritus (itch) is an aggravating sensation of the skin and/or mucous membranes^a^ commonly triggering an urge to scratch.	34/108 (31.5) [2;5]	NA
1c	Pruritus (itch) is an irritating sensation of the skin and/or mucous membranes^a^ commonly triggering an urge to scratch.	53/113 (46.9) [7;4]	NA
1d	Pruritus (itch) is an unpleasant and irritating sensation of the skin and/or mucous membranes^a^ commonly triggering an urge to scratch.	56/109 (51.4) [8;4]	NA
1e	Pruritus (itch) is a sensation of the skin and/or mucous membranes^a^ commonly triggering an urge to scratch.	48/107 (44.9) [9;5]	NA
2	Pruritus can be triggered, worsened or improved by a broad variety of external and internal factors.	85/107 (79.4) [8;2]	NA

	Definition of pruritus as a disease (*valid for chronic pruritus*)	1st Delphi, Total *n* = 120	2nd Delphi, Total *n* = 109
3	Chronic pruritus (CP) is categorically defined by the persistence of at least 6 weeks.	77/107 (72) [8;1]	NA
	Chronic pruritus is defined by the intermittent or continuous course over a period of at least 6 weeks.	NA	95/103 (92.2) [8;1]
4	Chronic pruritus can vary substantially in duration, intensity, quality, course, extent, location and impact on personal, social or professional quality of life and health.	90/107 (84.1) [8;1]	NA
5	Chronic pruritus differs from acute pruritus by long‐term structural and functional changes of itch processing.	72/106 (67.9) [8;2]	NA
	Chronic pruritus typically differs from acute pruritus by long‐term structural and functional changes of itch processing.	NA	88/102 (86.3) [8;0.25]
6	Pruritus may be a symptom of diseases.	80/105 (76.2) [9;2]	NA
7	Pruritus can persist independently despite resolution of the original disease or trigger and can become a disease in its own right (chronic pruritus).	83/106 (78.3) [9;2]	NA
8	Scratching is a physiological reaction to pruritus and can reduce the sensation.	72/106 (67.9) [8;2]	NA
	Scratching is a common reaction to both acute and chronic pruritus and can temporarily alleviate the sensation.	NA	88/102 (86.3) [8;1]
9	Chronic pruritus can be experienced as compulsive leading to aggravation and uncontrollable scratching.	66/106 (62.3) [8;2]	NA
	Chronic pruritus can lead to uncontrollable scratching and aggravation.	NA	91/102 (89.2) [8;1]
10	Chronic pruritus and scratching can trigger hedonic or rewarding sensations.	64/106 (60.4) [8;4]	NA
	Scratching may be experienced as pleasant and rewarding.	NA	81/101 (80.2) [9;1]
11	Prolonged itching and scratching might result in an itch‐scratch cycle.	88/106 (83) [9;1]	NA

*Note:* 1a‐1b variations of the same statement differing only in the underlined word. Green background: ≥75% agreement, yellow background: no consensus, entered in second Delphi round, red background: rejected variants of statement No. 1. Abbreviations: *n*, total number of voting participants per Delphi round; NA, not applicable; No. agreeing, number of participants agreeing (7–9 Likert scale range); No. voted, number of participants who voted per statement.

^a^ Skin and body openings such as the eyes and inner part of the mouth.

**FIGURE 3:**
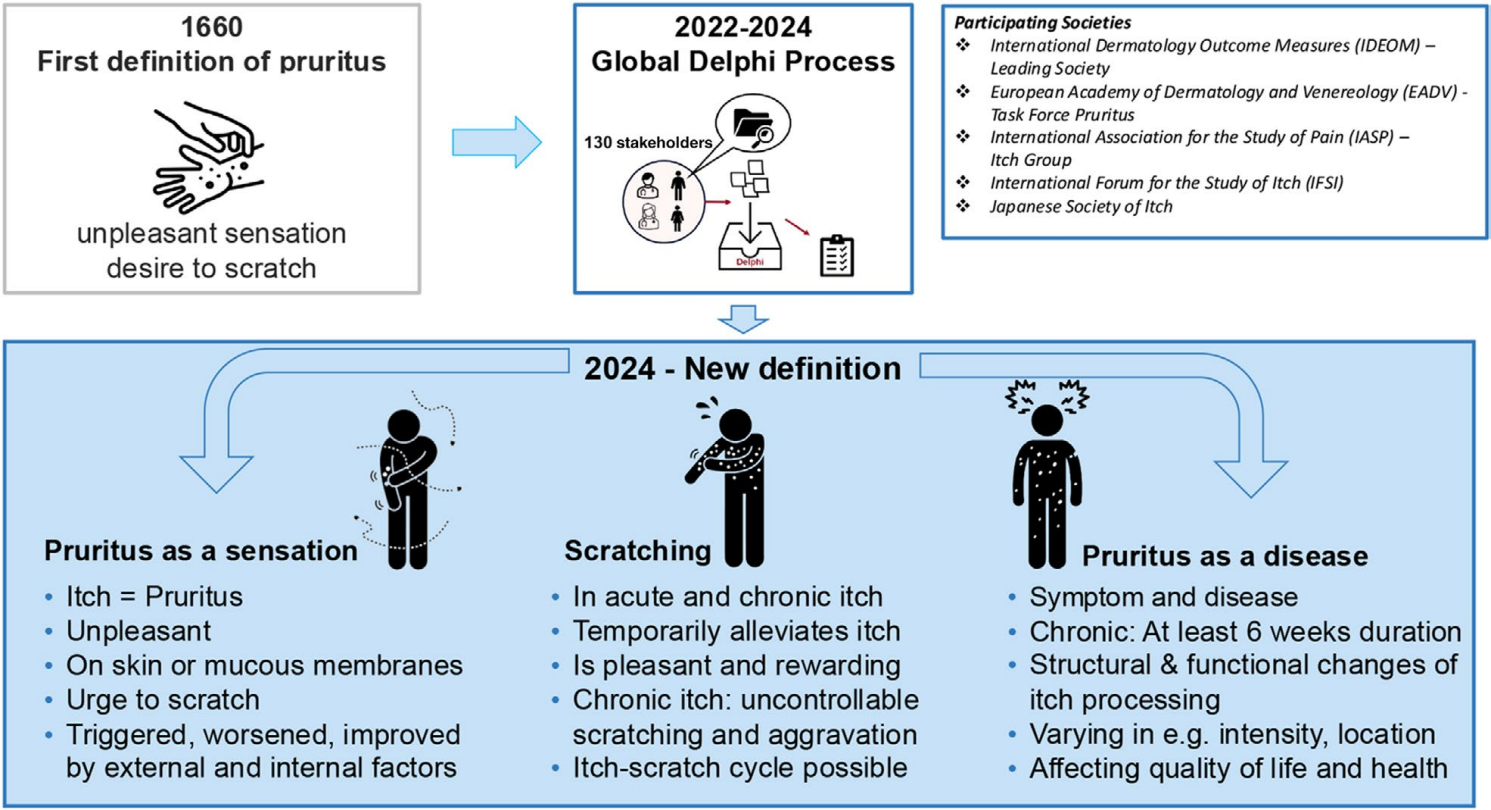
Evolution of the definition. In the early 17th century, the existence of skin diseases and the associated skin physiology began to be described.^11^ Samuel Haffenreffer was one of the first to attribute pruritus to the skin and even defined pruritus as a disease of the senses.^12^ He also defined pruritus as an unpleasant sensation that leads to the desire to scratch. This first definition, published in 1660, remained in use until September 2024, when the modern definition was presented for the first time at the Münster Pruritus Symposium. It separates the general sensory aspects of pruritus from the disease aspects of pruritus and specifies the characteristics of scratching.

After the first phase, 6/11 statements achieved a consensus and were closed (Table 1, Figure S1). This includes two statements defining the pruritus sensation and four defining pruritus as a disease. The steering committee analysed the comments on the remaining 5/11 statements and modified them where necessary. The revised statements and arguments were then opened again for Delphi voting. The participants were reinvited, receiving the statements, arguments and comments from the first round. All modified statements that entered the second Delphi round achieved consensus (Table 2, Figure 3). Voting results are shown in Table 1 and Figure S1.

**TABLE 2:** Definition of acute and chronic pruritus.

*Definition of pruritus as a sensation*. Pruritus (itch) is an unpleasant sensation of the skin and/or neighbouring mucous membranes commonly triggering an urge to scratch. Pruritus can be triggered, worsened or improved by a broad variety of external and internal factors.
*Definition of pruritus as a disease*. Chronic pruritus (CP) is defined by the intermittent or continuous course over at least 6 weeks. CP can vary substantially in duration, intensity, quality, course, extent, location and impact on personal, social or professional quality of life and health. CP typically differs from acute pruritus by long‐term structural and functional changes of itch processing. Pruritus may be a symptom of diseases. It can persist independently despite the resolution of the original disease or trigger and become a disease in its own right (chronic pruritus). Scratching is a common reaction to both acute and chronic pruritus and can temporarily alleviate the sensation. CP can lead to uncontrollable scratching and aggravation. Scratching may be pleasant and rewarding. Prolonged itching and scratching might result in an itch‐scratch cycle.

## DISCUSSION

This Delphi process succeeded in including global stakeholders and framing a novel definition for pruritus (Table 2). In line with the initiative's objective, the new definition addresses the expectations of the itch stakeholders, including patients. In multiple discussions, all aspects of pruritus were extensively debated from various perspectives, including those of clinicians and scientists from different societies, patients and health authorities. As a result, pruritus was defined with a focus on the human sensation and on disease aspects. This definition covers acute and chronic pruritus and their differences (Figure 3).

Interestingly, the definition of pruritus as a sensation, as presented in statement 1 (*Pruritus (itch) is an* *unpleasant* *sensation of the skin and/or neighbouring mucous membranes commonly triggering an urge to scratch*.), is not much different from the initial definition by Haffenreffer^1^ but is more precise as it also includes mucous membranes adjacent to the skin. In addition, it points to the fact that itch commonly triggers an urge to scratch, signifying that scratching is not always induced and not all patients succumb to the urge by actual scratching. All participants agreed that itch and pruritus can be used interchangeably and have the same meaning. The exact wording of the first statement regarding the type of sensation (*unpleasant/irritating/disruptive/aggravating*) provoked the most intense discussion as patients considered some suggested words as too weak to describe their suffering. However, it was concluded that as basic human sensations, pruritus and pain also act as ‘warning signs’ under physiological conditions, separating from relatively neutral context information provided by touch, vibration, warmth and cold. In this respect, it was argued that *unpleasant* serves well and is most suitable for describing the sensation as undesirable, offensive, repulsive and inducing negative emotions. *Aggravating* was rejected, for it could be misinterpreted as it refers to the conscious exaggeration of existing disease symptoms. Furthermore, *aggravating* points to a dynamism over time (worsening), which might not be the case in many forms of pruritus (e.g. acute pruritus). *Irritating* alone or in combination with *unpleasant* was rejected because tingling or tickling are irritating sensations, too. Thus, the word might not be sufficiently specific. Additionally, it can be misunderstood as ‘producing a skin lesion’. Finally, omitting an adjective was seen as lacking any direction about the nature of the itch.

The second statement (*Pruritus can be triggered, worsened or improved by a broad variety of external and internal factors*.) was deemed necessary to describe causal attributes of various origins, although the restriction to external or internal factors might not be self‐explanatory. External and internal factors that trigger, worsen or improve pruritus include chemical and physical factors, auditive, visual, neuronal, psychological and pharmacological aspects or contagious itch. Typical examples are histamine‐evoked itch (chemical factor) and warmth (physical factor), which typically worsen itch.

Regarding the definition of pruritus as a chronic disease in statement three, it was decided to maintain the previous definition^13^ of a time frame of 6 weeks as a separation between acute and chronic itch (C*hronic pruritus is defined by the intermittent or continuous course over a period of at least 6 weeks*.). Still, it became clear in the discussion that duration alone does not adequately reflect all aspects of CP pathophysiology and suffering.^8^, ^13^ In addition, it was agreed that there is currently no scientific basis for a transition to chronic itch after 6 weeks. However, clinically, a constant or intermittent (fluctuating) presence of pruritus for 6 weeks is of relatively long duration and not acute anymore. For practical purposes, it was decided to maintain the time frame of 6 weeks to enable an early starting point for diagnostics, targeting, for example, the early identification of malignant underlying entities, such as lymphoma and solid neoplasia, which, at early stages, are frequently asymptomatic except for the presence of itch.^14^

Statement four (*Chronic pruritus can vary substantially in duration, intensity, quality, course, extent, location and impact on personal, social or professional quality of life and health*.) reached an agreement at the first Delphi round. It clearly describes the characteristics of CP, including interindividual fluctuations and the induction of distress, which is the most significant individual effect.

Statement five (*Chronic pruritus typically differs from acute pruritus by long‐term structural and functional changes of itch processing*.) refers to the pathophysiological difference between acute and chronic itch, indicating that functional and structural changes occur in the nervous system, which lead to the persistence of the sensation independent of the original trigger.^15^ This statement in the new definition is critical as it indicates that patients´ suffering is qualitatively different from that in human experimental models with healthy volunteers. It underlines that CP is not simply the repetition of identical itch signals that eventually reach the 6‐week level and, therefore, would suffice the duration‐based definition. Instead, peripheral excitability changes (the level needed to activate nerve fibres to itch) facilitate ongoing activity in the itch system that, over time, changes the central processing of the itch signal. This neuroplasticity can underlie the severity and persistence of CP and has significant conceptual implications for basic science, such as limitations of acute itch models for the study of CP.^16^ As it cannot be ruled out that there may be CP states without such structural and functional changes, the word ‘typically’ was added.

Statements six (*Pruritus may be a symptom of diseases*.) and seven (*Pruritus can persist independently despite resolution of the original disease or trigger and can become a disease in its own right (chronic pruritus)*.), which summarize the clinical significance of statement five, achieved agreement at the first Delphi round. The statements explain the presence of pruritus as a disease symptom and as a disease itself.^17^ In statement seven, the course of pruritus in relation to the underlying origin is described. CP can persist independently of the stage or presence of the underlying aetiology. For example, it indicates CP if pruritus persists independently of therapy for the underlying aetiology. Further, a desynchronized stage of pruritus intensity and underlying disease severity points to CP as, for example, severe pruritus in mild to moderate atopic dermatitis (called itch‐dominant atopic dermatitis). Thus statement seven is especially expected to increase the understanding and awareness of CP and to open the path for health authority‐approved itch treatments and therapeutic guidelines.^18^

A unique feature of pruritus is scratching. Statements eight to *11 (8: Scratching is a common reaction to both acute and chronic pruritus and can temporarily alleviate the sensation. 9: Chronic pruritus can lead to uncontrollable scratching and aggravation. 10: Scratching may be experienced as pleasant and rewarding. 11: Prolonged itching and scratching might result in an itch‐scratch cycle*.) explain the role of scratching and how it promotes the chronicity of pruritus.^19^ Except for the last one, all statements needed clarification after the first round, for example, that acute and chronic pruritus induce scratching. Here, the participation of various stakeholders was essential for the outcome, as exemplified in the development of statements nine and 10. The designation of CP as compulsive led to misunderstanding, and statement 9 was rejected. It was discussed that a reward circuit is involved with pruritus and scratching. Still, the association with compulsive behaviour can also confuse as it implies negative aspects of behaviour with an incorrect connotation. Accordingly, the sentence was simplified without a negative connotation and received high agreement. In statement 10, the word ‘hedonic’ in its first version was hard to understand, leading to the second version of this statement, where scratching is described in patients' words as a ‘pleasant’ experience. Scratching, as used here, refers to the mechanical habit of eliminating itch, such as rubbing, tearing, pressing with a pointed object and other types of touch. Thus, it is assumed that scratching is a broad enough word covering many possible reactions to itch and similar sensations.^18^, ^20^

With this Delphi process, we achieved alignment on an updated definition of pruritus that aims to support practical use in a healthcare setting, therapy development and communication with patients, health authority access, insurance companies and other stakeholders. The updated definition should help recognize pruritus as a multidisciplinary severe symptom not primarily attributed to parasitoses or psychiatric entities such as compulsive and delusional disorders.

The chosen approach with a pre‐Delphi phase was critical for the Delphi quality and success of the consensus‐building. The approach allowed for debate, alignment and incorporation of expert and patient insights and, accordingly, successful definition of up‐to‐date statements that entered the Delphi process. The presentation of the pro and con arguments in the Delphi questionnaire facilitated well‐informed voting. The slight difference in the number of voting panel members between the two rounds is not expected to have influenced the outcome. The mode and IQR values confirm the strength of the agreement. The participation of multiple societies allowed a broad representation of stakeholders from 35 countries. The surveys were performed in English, limiting participation in the event of language hurdles.

As next steps, the new definition will be carefully translated into multiple languages and then disseminated through guideline committees, networks of itch and a follow‐up publication.

## CONCLUSIONS

This Delphi process led to an international, multidisciplinary consensus on an updated definition of pruritus, which better reflects the complexity of the sensation, including symptoms and time course, as well as emotional impact and causal aspects.

The new definition separately addresses itch sensation and disease. While the first part clarifies the original definition and relates mainly to acute pruritus, the characterization of CP as a disease is new and significant. It separates the sensation from the initial cause, considering it an independent disease, as it has already been implemented for pain. Pruritus is devastating, and the new definition sets out to facilitate healthcare, communication with decision‐making bodies, and, importantly, the first step towards researching and developing suitable treatments, including targets beyond the initial triggers.

## AUTHOR CONTRIBUTIONS

All authors contributed substantially to the study's conception and/or design, data analysis and interpretation. They also drafted and critically revised the article for important intellectual content and gave final approval of the paper. In addition, Henning Wiegmann organized the data acquisition.

## FUNDING INFORMATION

International Dermatology Outcome Measures (IDEOM) and the German Research Foundation (DFG—Deutsche Forschungsgemeinschaft; grant FOR 2690 to MS and SST, grant FOR 5211 to SST).

## CONFLICT OF INTEREST STATEMENT

Sonja Ständer: was speaker and/or consultant and/or investigator and/or has received research funding from Almirall S. A., Amgen Inc., Amgen Europe GmbH, Celldex Therapeutics Inc., Clexio Biosiences Ltd., Focus‐Insight Healthtech Group Co. Ltd., Galderma Laboratorium GmbH, Galderma R&D LLC., Galderma S.A., GSK R&D Ltd., Incyte Corporation, Klirna Biotech Inc., Leo Pharma, Lilly Deutschland GmbH, Novartis Pharma GmbH, Sanofi‐Aventis Deutschland GmbH, Sanofi‐Aventis R&D, Sanofi Genzyme Corporation, TouchIME, Vifor Pharma Deutschland GmbH P. G. Unna Academy, Martin Schmelz: was consultant for Merz AG, Lilly, Bayer, Medtronic. Ethan Lerner: was on the Scientific Advisory Board of Escient Pharmaceuticals. Hiroyuki Murota: was speaker and/or consultant and/or investigator of Leo Pharma, Lilly, Novartis Pharma, AbbVie, Sanofi, Pfizer, Maruho Co. Ltd., Torii pharma, Otsuka pharma, Sun pharma. Leigh Nattkemper: consultant and/or investigator and/or has received research funding from Celldex Therapeutics, Kane Biotech and Galderma. Adam Reich: was speaker and/or consultant and/or investigator and/or has received research funding from Abbvie, Almirall, Alumis, Alvotec, Amgen, AnaptysBio, Arcutis, Argenx, Bausch Health, Biocon, Bioderma, Biothera, BMS, Boehringer Ingelheim, Celgene, Celldex Therapeutics, CellTrion, Chema Elektromet, CuraTeq Biologics, Dice Therapeutics, Eli Lilly, Galderma, Horizon, Incyte Corporation, Janssen, Kiniksa, Leo Pharma, Medac, Nektar, Novartis, Numab, Pfizer, Pierre Fabre, Sandoz, Sanofi, Takeda, Trevi, UCB. Lea‐Sophie Stahl: is an advisor for Galderma. Gil Yosipovitch: was speaker and/or consultant and/or investigator and/or has received research funding from Abbvie, Arcutis, Almiral, Amgen, Celldex, Clexio, Escient Pharmaceuticals, Eli Lilly, Galderma, GSK, Kamari, LEO Pharma, Merck, Novartis, Pfizer, Pierre Fabre, Regeneron Pharmaceuticals, Inc., Sanofi, Vifor. Elke Weisshaar: was/is a consultant and/or investigator and/or received research money from Galderma, German Research Foundation (DFG), German Statutory Insurance (DGUV), Kiniksa, Leo Pharma, Sanofi and Trevi. Henning Wiegmann: none. Christian Apfelbacher: Has received institutional funding from the Dr. Wolff Group and Bionorica SE, and consultancy fees from the Dr. Wolff Group, Bionorica SE, Sanofi, Incyte Biosciences, RHEACELL, Pfizer, EFCNI, LEO Pharma and IVDK. Brian S. Kim: is co‐founder of Alys Pharmaceuticals; he has served as a consultant for 23andMe, ABRAX Japan, AbbVie, Amgen, Attovia Therapeutics, Cara Therapeutics, Clexio Biosciences, Eli Lilly and Company, Escient Pharmaceuticals, Evommune, Galderma, LEO Pharma, Micreos, Novartis, Pfizer, Recens Medical, Regeneron, Sanofi, Septerna, Teva, Trevi Therapeutics, Triveni Bio; he has stock in ABRAX Japan, Alys Pharamaceuticals, Attovia Therapeutics, Locus Biosciences, Recens Medical and Triveni Bio; he holds a patent for the use of JAK1 inhibitors for chronic pruritus.

## ETHICAL APPROVAL

Reviewed and approved by the IRB of Westfalian‐Lippe, Münster, Germany; approval No. 2024‐045‐f‐S.

## ETHICS STATEMENT

The patients in this manuscript have given written informed consent to publish their case details.

## Supporting information


**Figure S1.** Detailed results of each statement from both Delphi rounds.

**Table S1.** Participants of the Delphi (A) and pre‐Delphi (B) process (alphabetical order).

## Data Availability

The data supporting this study's findings are available from the corresponding author upon reasonable request.
