# Supplementary figures and images for: A multidisciplinary Delphi consensus on the modern definition of pruritus: Sensation and disease
Source: J Eur Acad Dermatol Venereol. 2025 Jul 17;40(1):59–66. doi: 10.1111/jdv.20851 (PMC12512202; doi:10.1111/jdv.20851)

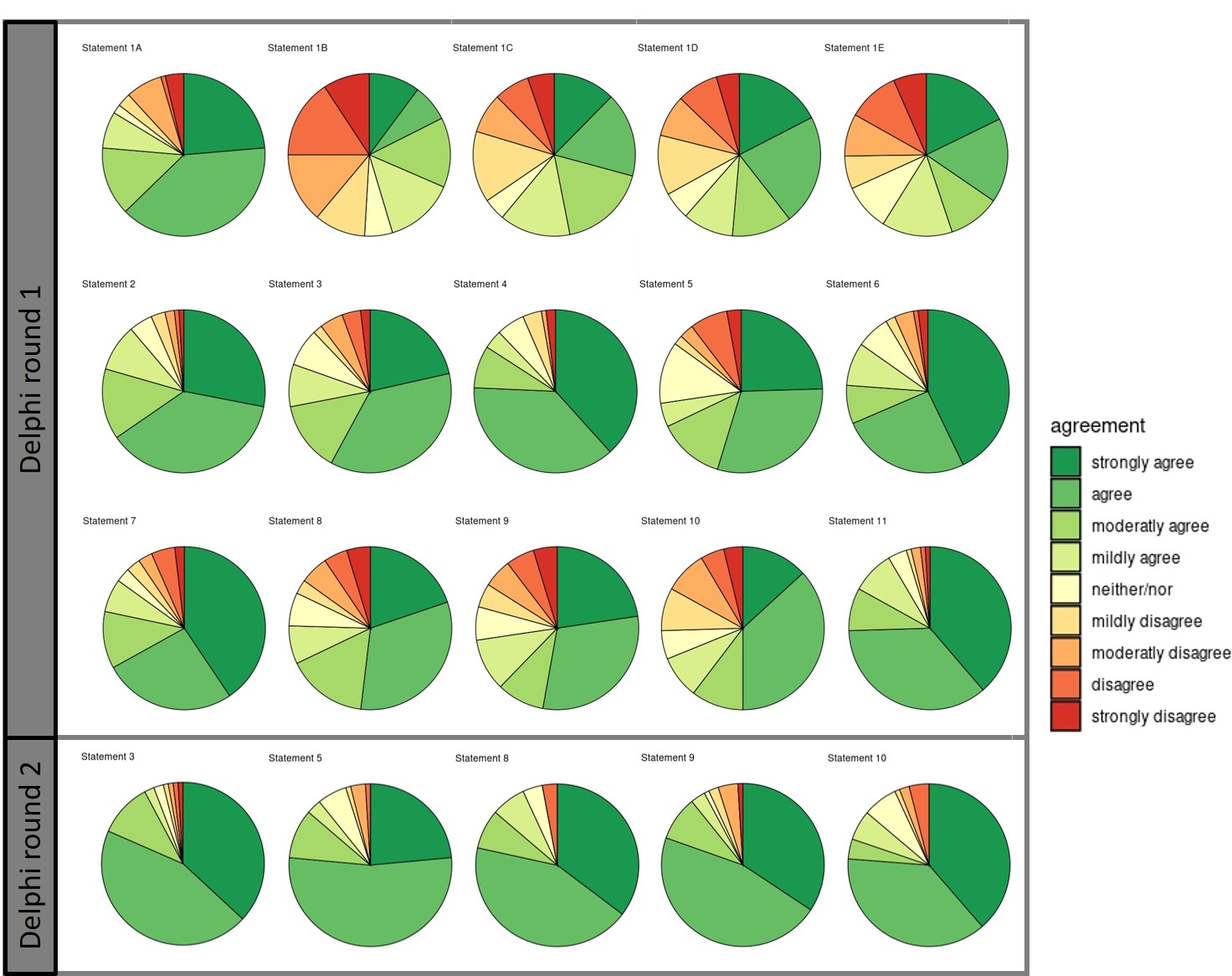

Supplement: Supplementary file 1 — Figure S1. Detailed results of each statement from both Delphi rounds. [file JDV-40-59-s002.jpg]
