# Supplementary material for: A multidisciplinary Delphi consensus on the modern definition of pruritus: Sensation and disease
Source: J Eur Acad Dermatol Venereol. 2025 Jul 17;40(1):59–66. doi: 10.1111/jdv.20851 (PMC12512202; doi:10.1111/jdv.20851)
Supplement: Supplementary file 2 — Table S1. Participants of the Delphi (A) and pre‐Delphi (B) process (alphabetical order). [file JDV-40-59-s001.docx]

**eTable 1 Participants of the Delphi (A) and Pre-Delphi (B) process (alphabetical order)**

1. **Delphi Stage Participants**

| **Last name** | **First name** | **Country** |
| --- | --- | --- |
| Abuabara | Katrina | USA |
| Agelopoulos | Konstantin | Germany |
| Akiyama | Tasuku | USA |
| Ali | Javeria | USA |
| Andrade | Luis | USA |
| Aoki | Valeria | Brazil |
| Arshad | Samreen | USA |
| Auyeung | Kelsey | USA |
| Barta | Kelly | USA |
| Becker | Jennifer | Netherlands |
| Bewley | Anthony | United Kingdom |
| Biazus Soares | Georgia | USA |
| Bird | Mick | USA |
| Bobko | Svetlana | Russia |
| Bollemeijer | Juliette | Netherlands |
| Brenaut | Emilie | France |
| Brooks | Sarah | USA |
| Butler | Daniel | USA |
| Butts Snowden | Kim | Not known |
| Calvo | Margarita | Chile |
| Chambenoit | Oliver | Switzerland |
| Chamli | Amal | Tunis |
| Chisolm | Sarah | USA |
| Choi | Ellie | Singapore |
| Davidson | Steve | USA |
| Ding | Delaney | USA |
| Ebata | Toshiya | Japan |
| Ehlert | Athena | Not known |
| Elberling | Jesper | Denmark |
| Elmariah | Sarina | USA |
| Evers | Andrea | Netherlands |
| Fedder | Nancy | USA |
| FitzGerald | Allison | Canada |
| Fleischer | Alan | USA |
| Forte | Jo-Anne | Not known |
| Garcovich | Simone | Italy |
| Gonçalo | Margarida | Portugal |
| Gonzalez | Tayler | USA |
| **Last name** | **First name** | **Country** |
| Gottlieb | Alice | USA |
| Greene | Meloody | United Kingdom |
| Hachisuka | Junichi | United Kingdom |
| Halvorsen | Jon Anders | Norway |
| Hashimoto | Takashi | Japan |
| Heine | Lena | Germany |
| Hensel | Patrick | Switzerland |
| Hurtová | Tatiana | Slovakia |
| Ikoma | Akihiko | Japan |
| Irvine | Alan | Ireland |
| Ishiuji | Yozo | Japan |
| Jurcakova | Danica | Bratislava |
| Kim | Brian | USA |
| Kirk | Charmaine | Canada |
| Ko | Hon-Sum | USA |
| Kremer | Andreas | Switzerland |
| Kupfer | Joerg | Germany |
| Lambert | Julien | Belgium |
| Lavrukhin | Oleg | USA |
| Le Garrec | Raphaele | France |
| Lebwohl | Mark | USA |
| Legat | Franz | Austria |
| Lerner | Ethan | USA |
| Levy | Stephane | Not known |
| Luke | Markham | USA |
| Lynde | Charles | USA |
| Malyshkina | Anna | Germany |
| Manjunath | Jaya | USA |
| Mcglone | Francis | United Kingdom |
| Meltzer | Rachel | USA |
| Mettang | Thomas | Germany |
| Metz | Martin | Germany |
| Misery | Laurent | France |
| Mittal | Asit | India |
| Mowla | Mohammad Rafiqul | Bangladesh |
| Müller | Simon | Switzerland |
| Müller | Svenja | Germany |
| Murota | Hiroyuki | Japan |
| Nakahara | Takeshi | Japan |
| Nattkemper | Leigh | USA |
| Noda | Aliene | Switzerland |
| Obreja | Otilia | Schweiz |
| **Last name** | **First name** | **Country** |
| Olteanu | Rodica | Romania |
| Padberg-White | Greta | USA |
| Papadavid | Evangelia | Greece |
| Paul | Julia | USA |
| Pereira | Manuel | Germany |
| Poskanzer | Kira | USA |
| Qureshi | Aamir | USA |
| Reich | Adam | Poland |
| Rhodes | Lisa | USA |
| Ring | Johannes | Germany |
| Ringkamp | Matthias | USA |
| Riquelme Mc Loughlin | Constanza | Spain |
| Romanov | Dmitry | Russia |
| Salphale | Pankaj | United Kingdom |
| Satoh | Takahiro | Japan |
| Savk | Ekin | Turkey |
| Schalk | Erin | USA |
| Schmelz | Martin | Germany |
| Schut | Christina | Germany |
| Semenov | Yevgeniy | USA |
| Serra-Baldrich | Esther | Spain |
| Smith Begolka | Wendy | USA |
| Solinski | Jürgen | Germany |
| Spicer | Olesya | USA |
| Stahl | Lea-Sophie | Germany |
| Ständer | Hartmut | Germany |
| Ständer | Sonja | Germany |
| Stefaniak | Aleksandra | Poland |
| Stephen | John | India |
| Strand | Vibeke | USA |
| Streit | Markus | Switzerland |
| Summers | Danine | USA |
| Szepietowski | Jacek | Poland |
| Takamori | Kenji | Japan |
| Takanami | Keiko | Japan |
| Tominaga | Mitsutoshi | Japan |
| Tran | Thanh Nga | USA |
| Turkey | Lyla | USA |
| van de Burgt | Emmy | Netherlands |
| van Laarhoven | Antionette | Netherlands |
| Ver Heul | Aaron | USA |
| **Last name** | **First name** | **Country** |
| Wallengren | Joanna | Sweden |
| Weisshaar | Elke | Germany |
| Wheeler | Josh | USA |
| Whitmore | Lyn | Australia |
| Xie | Lingxiao | USA |
| Xu | Steve | USA |
| Yosipovitch | Gil | USA |
| Yu | Huasheng | USA |
| Zeidler | Claudia | Germany |

1. **Pre-Delphi Stage Participants**

| **Last name** | **First name** | **Country** |
| --- | --- | --- |
| Apfelbacher | Christian | Germany |
| Arshad | Samreen | USA |
| Bauer | Martin | Germany |
| Bobko | Svetlana | Russia |
| Brenaut | Emilie | France |
| Buddhadev | Dr Rajesh | India |
| Calvo | Margarita | Chile |
| Cirulli | Josh | USA |
| Davidson | Steve | USA |
| Del Mestre | Pablo | Argentina |
| Ebata | Toshiya | Japan |
| Elberling | Jesper | Danmark |
| Evers | Andrea | Netherlands |
| Goncalo | Margarida | Portugal |
| Hachisuka | Junichi | UK |
| Hensel | Patrick | Switzerland |
| Hoon | Mark | USA |
| Huang | Adam | Taiwan |
| Ikoma | Akihiko | Japan |
| Irvine | Alan | Ireland |
| Ishiuji | Yozo | Japan |
| Jensen | Dane | USA |
| Kim | Brian | USA |
| Kremer | Andreas | Switzerland |
| Kupfer | Jörg | Germany |
| Kwatra | Shawn | USA |
| Legat | Franz | Austria |
| Lerner | Ethan | USA |
| Mcglone | Francis | United Kingdom |
| Metz | Martin | Germany |
| Misery | Laurent | France |
| Mittal | Asit | India |
| Mohawk | Jennifer | USA |
| Mowla | Mohammad Rafiqul | Bangladesh |
| Müller | Simon | Switzerland |
| Murota | Hiroyuki | Japan |
| Nakahara | Takeshi | Japan |
| Noda | Aliene | Switzerland |
| Obreja | Otilia | Schweiz |
| Pereira | Manuel | Germany |
| Papadavid | Evangelia | Greece |
| **Last name** | **First name** | **Country** |
| Reich | Adam | Poland |
| Rhodes | Lisa | USA |
| Ring | Johannes | Germany |
| Ross | Sarah | USA |
| Salphale | Pankaj | United Kingdom |
| Satoh | Takahiro | Japan |
| Şavk | Ekin | Turkey |
| Schmelz | Martin | Germany |
| Schneider | Gudrun | Germany |
| Schut | Christina | Germany |
| Serra-Baldrich | Esther | Spain |
| Sharif | Behrang | Canada |
| Smith Begolka | Wendy | USA |
| Spruijt | Odette | Australia |
| Ständer | Hartmut | Germany |
| Ständer | Sonja | Germany |
| Stephen | John | India |
| Svenja | Müller | Germany |
| Szepietowski | Jacek | Poland |
| Tomlinson | Amelia | USA |
| van de Burgt | Emmy | Nederland |
| Wallengren | Joanna | Sweden |
| Weisshaar | Elke | Germany |
| Wiggins | Simmi | United Kingdom |
| Winkelman | Warren | USA |
| Yosipovitch | Gil | USA |
| Zeidler | Claudia | Germany |
| Zuo | Ying | China |
